# In Memorium—Dr. Kulp

**Published:** 1895-02

**Authors:** 


### In Memory of Dr. Kulp.

At a call meeting of the Des Moines Dental Society held in the office of Dr. G. W. Miller, January 14, 1895, at which Drs. S. L. Edwards, A. R. Begun, G. W. Miller, C. Thomas, T. A. Hallet, G. W. Fuller, J. B. Entrikin, Jessie M. Ritchey and H. N. Edwards were present, Dr. C. Thomas reported the receipt of a telegram announcing the death of Dr. W. O. Kulp, of Davenport, Iowa. A floral pillow was ordered for the funeral and a committee appointed to prepare appropriate resolutions.

Dr. Jesssie M. Ritchey read a poem in memory of Dr. W. O. Kulp, which on request was granted for publication.

At the adjourned meeting held in the office of Dr. Begun on Tuesday, January 22, the report of Committee on Resolutions was adopted as follows:

As it has pleased our Heavenly Father to call from labor to rest our esteemed brother, Dr. W. O. Kulp, we, the members of the Des Moines Dental Society, desire to express our sorrow for the loss of one whose life was ever devoted to the welfare of his profession and extend to the bereaved family our sincere sympathy in their deep affliction.

*Resolved*, That in his death the profession has lost a most worthy member and the Dental Department of our State University an earnest and faithful instructor.

THOS. A. HALLETT,
SURRY L. EDWARDS,
JESSIE M. RITCHEY,
Committee.
HORACE N. EDWARDS,
Secretary.

Lines written by Dr. Jessie M. Ritchey in memory of Dr. W. O. Kulp:

Death thrust the sickle in
  The ripened grain doth fall;
We are awed and stilled by the thought
  Thus will He garner us all.

We know that our friend was noble,
  Great-hearted and true and brave;
O, why in the prime of manhood,
  Has he left us now for the grave?

Did not life with all its lessons
  Have missions for him to do?
Was it that in skill and cunning
  His hands were palsied, untrue?

No, like the oak in the forest
  With its branches that herald the sun,
Which, in its strength and its power
  Is stricken and lieth dumb.

So our brother in nature majestic
  At the time of life's high tide,
At the crown of manhood's rich glory,
  Has passed to the other side.

O! Death where is thy sting
  O! where will thy victory be
If from life's fullest fruition
  He is brought to eternity?

If from striving and yearning
  For truth, for supremest skill,
He has closed earth's eyes to enter
  The revealments of the invisible?

For on earth with vision so feeble,
  We falter from day to day
In the mists and shadows surrounding
  That darken and dim our way.

Till with life we are a wearied
  And with care our souls are done,
  Then earth will close to the spirit
As the day doth close with the sun.

But the morn will rise resplendent
  And the clouds which covered the night
Will be burst and cast asunder
  By the dawn of eternal night.

